# Basic symptoms – lessons from Spanish-speaking countries

**DOI:** 10.1192/j.eurpsy.2025.179

**Published:** 2025-08-26

**Authors:** N. Jimeno

**Affiliations:** 1 Psychiatry, University of Valladolid; 2 Research Group on Clinical Neuroscience of Castile and Leon, Valladolid, Spain

## Abstract

**Abstract:**

The main contributions of Spanish and Hispano-American authors to the study of basic symptoms, either published in Spanish and English, will be briefly presented. Former publications mainly included translation and validation of different versions of the Frankfurt Complaint Questionnaire, transversal and longitudinal studies, and their role for psychosocial rehabilitation, also in a Penitentiary Psychiatric Hospital. More recent research includes the network analysis of basic, attenuated, and frank psychotic symptoms on 460 subjects attending a German early detection service, where disorganized communication, delusions and hallucinations were the most central symptoms. Interestingly, cognitive and perceptual disturbances, included in basic symptom criteria, appeared to develop across attenuated symptoms to frank positive psychotic symptoms. Concerning the finding of three clusters of symptoms, **“**subjective disturbances**”**, **“**positive symptoms and behaviors**”**, and **“**negative and anxious-depressive symptoms**”**, the predominately attenuated hallucinations of both SIPS and PANSS joined the basic symptoms in **“**subjective disturbances**”**, therefore underlining the importance of insight in separating true psychotic hallucinations from other hallucinatory experiences and not justifying antipsychotic medication.

**Disclosure of Interest:**

None Declared

